# Growth hormone-secreting pituitary adenoma combined with Graves’ disease: retrospective case series and literature review

**DOI:** 10.1530/EC-23-0439

**Published:** 2024-03-04

**Authors:** Caiyan Mo, Tao Tong, Ying Guo, Zheng Li, Liyong Zhong

**Affiliations:** 1 Department of Endocrinology, Beijing Tiantan Hospital, Capital Medical University, Beijing, China

**Keywords:** growth hormone-secreting pituitary adenoma, Graves' disease, growth hormone, insulin-like growth factor 1, thyroid hormone

## Abstract

**Purpose:**

The coexistence of growth hormone-secreting pituitary adenoma (GHPA) and Graves' disease (GD) is rare. This study aimed to investigate the relationship between growth hormone (GH)/insulin-like growth factor 1 (IGF-1) levels and thyroid function in patients with GHPA combined with GD and to explore the underlying mechanisms.

**Methods:**

Eleven patients with GHPA combined with GD during 2015-2022 were collected by searching the medical record system of Beijing Tiantan Hospital, Capital Medical University. Changes in GH/IGF-1 levels and thyroid function were compared before and after the application of antithyroid drugs (ATD) and before and after transsphenoidal surgery (TSS) or somatostatin analog (SSA) treatment, respectively.

**Results:**

After the application of ATD, with the decrease of thyroid hormone levels, GH/IGF-1 levels also decreased gradually. In patients without ATD application, after surgery or SSA treatment, thyroid hormone levels decreased as GH/IGF-1 decreased.

**Conclusion:**

Hyperthyroidism due to GD promotes the secretion of GH/IGF-1, and when thyroid hormone levels were decreased by the use of ATD, GH and IGF-1 levels were also decreased, suggesting that thyroid hormones may influence the synthesis and secretion of GH/IGF-1. The use of ATD to control thyrotoxicosis before TSS is not only beneficial in reducing the risk of anesthesia but may help to promote biochemical control of GHPA. On the other hand, high levels of GH/IGF-1 in patients with GHPA also exacerbate GD hyperthyroidism, which is ameliorated by a decrease in GH/IGF-1 levels by TSS or SSA treatment, suggesting that the GH–IGF-1 axis promotes growth, thyroid function, and thyroid hormone metabolism.

## Introduction

Growth hormone-secreting pituitary adenoma (GHPA) is due to the overproduction of growth hormone (GH) by pituitary adenomas, which stimulates the liver to produce insulin-like growth factor 1 (IGF-1), and the long-term overproduction of GH and IGF-1 promotes the overgrowth of soft tissues, bones, and cartilages throughout the body, leading to a series of typical signs and symptoms of acromegaly or gigantism, and can cause multi-organ/system complications such as respiratory, cardiovascular, digestive system, and glucose metabolism. The global prevalence of GHPA has been reported to be 5.9 (95% confidence interval (CI): 4.4–7.9) per 100,000 persons, while the incidence rate was 0.38 (95% CI: 0.32–0.44) cases per 100,000 person-years (1). Due to the insidious symptoms, the average time from symptom onset to diagnosis is usually about 7–10 years. The average life expectancy of patients with GHPA is shortened by more than 10 years and mortality is doubled compared to the general population (2).

Graves' disease (GD) is an autoimmune disease of the thyroid gland and is the most common cause of hyperthyroidism. The incidence is 20–50 cases per 100,000 people, with a peak between the ages of 30 and 50. The binding of thyroid stimulating hormone receptor antibodies (TRAb) to thyroid-stimulating hormone (TSH) receptors leads to unregulated thyroid hormone production independent of pituitary TSH, resulting in hyperthyroidism (3). GD can also present with eyelid retraction, proptosis, and ocular motility disorders, and is known as Graves' ophthalmopathy (GO), with an incidence of 2.9/100,000/year in men and 16/100,000/year in women, resulting in visual impairment and reduced quality of life (4).

The thyroid structure and function are affected in patients with GHPA. Goiter has been reported in 18–50% of patients with acromegaly, and 5% of patients with significant hyperthyroidism, most of whom have non-autoimmune hyperthyroidism (5, 6). Despite the increased incidence of thyroid disease in patients with GHPA, the coexistence of GHPA with GD remains very rare. In a study of 129 patients with acromegaly, 93 (72.1%) patients with acromegaly had thyroid disease, with combined GD in about 4.5% (7). It is unclear whether the coexistence of GHPA and GD will have an impact on the condition and treatment of patients. Therefore, this study collected patients with GHPA combined with GD in our center and reviewed previously published cases to investigate the relationship between GH/IGF-1 levels and thyroid function in dual disease coexistence cases and to explore the possible mechanisms involved.

## Materials and methods

### Study population and design

Beijing Tiantan Hospital, Capital Medical University, is one of the largest pituitary care centers in Asia. By searching the subject terms ‘growth hormone-secreting pituitary adenoma’ or ‘acromegaly’ and ‘Graves' disease’ or ‘diffuse toxic goiter’ in the medical record system, we finally retrieved a total of 11 patients with combined GD out of 625 patients with GHPA. The diagnostic criteria of GHPA included: clinical features of gigantism or acromegaly; unsuppressed serum GH to less than 1 ng/mL after administration of a glucose load; elevated gender- and age-matched IGF-1 levels; and pituitary adenoma diagnosed by contrast-enhanced magnetic resonance imaging (MRI) and confirmed by postoperative pathology as GHPA. The diagnostic criteria of GD included: symptoms and signs of hypermetabolism due to thyrotoxicosis, elevated serum thyroid hormone levels and suppressed TSH, diffuse enlargement of the thyroid gland, and TRAb are positive.

### Laboratory methods

Fasting serum GH, IGF-1, total triiodothyronine (TT3), total thyroxine (TT4), TSH, free triiodothyronine (FT3), free thyroxine (FT4), and TRAb levels were measured between 07:00 and 09:00 h by chemiluminescence immunoassay (IMMULITE 2000 Immunoassay System, UniCel DxI 800 Access Immunoassay System, respectively). We recorded the patients' gender, age, duration of disease (including the time from symptom onset to inclusion in this study), and the maximum diameter (Dmax) of the pituitary tumor on the contrast-enhanced head MRI scan. The height and weight of the patients were also recorded, and the body mass index (BMI) was calculated by dividing the weight (kg) by the square of the height (m^2^). The reference range for GH is 3 ng/mL or lower, and the upper limit of the measurement range is 40 ng/mL. The reference range for IGF-1 was obtained from the Chinese reference intervals. The reference ranges for TT3, TT4, TSH, FT3, FT4, and TRAb were 1.01–2.48 nmol/L, 69.97–152.52 nmol/L, 0.49–4.91 μIU/mL, 3.28–6.47 pmol/L, 7.64–16.03 pmol/L, and 0–1.7 IU/L, respectively.

## Results

### Case series

A total of 11 patients with GHPA combined with GD were included, containing six males and five females, as shown in Table 1. All patients presented with acromegaly except for case 11 who presented with overgrowth and tall stature. All patients presented with thyrotoxicosis and case 8 also had mild bilateral exophthalmos. Of these 11 patients, eight patients underwent pituitary tumor resection in our hospital, including cases 1, 2, 3, 6, 7, 8, 10, and 11, while cases 4 and 5 were treated surgically at other hospitals, and all postoperative pathology of the patients showed pituitary adenoma and immunohistochemistry showed GH positive. The pituitary tumor in case 9 is a microadenoma and is currently treated with regular monthly SSA rather than TSS. Except for case 11, in which the age of onset was 15 years, all had a young or middle-aged onset, and the median age was 34 (25–42) years. GHPA has an insidious onset and is diagnosed with a relatively long course of the disease, and the head MRI shows usually a macroadenoma. Similarly, in the present study, the median duration of disease was 36 (8–116) months, and the median Dmax was 17.00 (12.00–25.00) mm. All patients had elevated pretreatment levels of GH and IGF-1, with a median of 5.91 (5.09–40) ng/mL for GH and 790 (586–1112) ng/mL for IGF-1. All thyroid functions of the patients showed elevated thyroid hormones, suppressed TSH levels, and positive TRAb. The median FT3, FT4, TSH, and TRAb were 10.27 (7.37–16.63) pmol/L, 24.44 (21.41–34.91) pmol/L, 0.012 (0.005–0.060) μIU/mL, and 6.56 (2.98–9.30) IU/L, respectively.

**Table 1:** Baseline characteristics of patients.

Patient	Age (year)/gender	Disease duration (month)	Clinical manifestations	BMI (kg/m^2^)	Dmax (mm)	Pre-GH (ng/mL)	Pre-IGF (ng/mL)	Pre-TT3 (nmol/L)	Pre-TT4 (nmol/L)	Pre-TSH (μIU/mL)	Pre-FT3 (pmol/L)	Pre-FT4 (pmol/L)	Pre-TRAb (IU/L)	Treatment	Post-GH (ng/mL)	Post-IGF (ng/mL)	Post-TT3 (nmol/L)	Post-TT4 (nmol/L)	Post-TSH (μIU/mL)	Post-FT3 (pmol/L)	Post-FT4 (pmol/L)	Post-TRAb (IU/L)
1	43/M	120	Acromegaly, thyrotoxicosis	25.65	34	40	1112	2.73	144.51	0.012	6.56	22.49	1.77	SSA, TSS	20.5	425	1.13	82.9	0.024	3.59	13.71	−
2	36/F	72	Acromegaly, thyrotoxicosis	27.1	17	40	1321	3.41	161.6	0.002	10.27	25.34	8.75	ATD, TSS, SSA	2.87	1146	2.04	115.16	0.002	5.91	17.52	−
3	42/F	36	Acromegaly, thyrotoxicosis	23.9	14	5.13	778	4.37	210.96	0.027	16.63	29.73	9.3	ATD, TSS	2.84	767	3.3	168.18	0.016	10.65	24.83	−
4	42/F	122	Acromegaly, thyrotoxicosis	28.96	18	5.27	586	4.83	233.04	0.001	15.76	34.91	5.17	ATD, TSS	0.203	145	1.3	92.29	0.02	4.97	9.05	1.18
5	52/M	6	Acromegaly, thyrotoxicosis	24.22	12	3.39	947	2.85	162.64	0.214	7.37	23.36	6.62	SSA, TSS	1.21	224	1.36	115.51	0.095	4.3	15.08	−
6	21/F	8	Acromegaly, thyrotoxicosis	24.77	48	5.91	728	7.45	350.78	0.005	26.64	62.82	5.17	SSA, TSS	1.93	417	0.84	93.99	0.058	3.92	8.46	12.37
7	32/M	116	Acromegaly, thyrotoxicosis	27.59	20	7.78	534	2.23	224.15	0.319	5.11	17.04	6.56	SSA, TSS	1.23	399	2.66	206.15	1.515	5.66	12.31	−
8	30/F	24	Acromegaly, thyrotoxicosis, proptosis	20.96	25	40	790	2.56	194.67	0.06	7.48	21.41	2.98	SSA, TSS	5.21	714	1.47	154.64	0.932	6.58	14.86	2.3
9	34/M	48	Acromegaly, thyrotoxicosis	28.41	7	4.05	545	3.59	141.85	0.005	12.56	24.44	2.7	ATD	1.31	216	1.14	58.6	3.822	4.38	7.16	1.05
10	25/M	21	Acromegaly, thyrotoxicosis	26.54	6	5.09	1019	2.53	128.33	0.02	8.53	17.23	10.87	SSA, TSS	0.859	945	1.64	53.36	1.697	4.58	7.49	−
11	15/M	7	Overgrowth, thyrotoxicosis	24.93	12	10.2	1126	7.21	335.95	0.005	29.3	60.65	12.34	ATD, TSS	1.91	582	2.53	90.18	0.077	6.57	9.53	−

ATD, antithyroid drugs; BMI, body mass index; Dmax, maximum diameter; F, female; FT3, free triiodothyronine; FT4, free thyroxine;GH, growth hormone; IGF-1, insulin-like growth factor 1; M, man; SSA, somatostatin analog; TRAb, thyroid-stimulating hormone receptor antibodies; TSH, thyroid-stimulating hormone; TSS, transsphenoidal surgery; TT3, total triiodothyronine; TT4, total thyroxine;.

Among the included patients, cases 2, 3, 4, 9, and 11 were treated with ATD preoperatively because thyrotoxicosis could increase the anesthetic risk of surgery. Changes in thyroid function after ATD treatment and before treatment with TSS or SSA are shown in Table 1. The median duration of ATD treatment was 6 (1–9) months. After the use of ATD, the levels of TT3, TT4, FT3, and FT4 were all decreased, and the levels of GH and IGF-1 were also significantly decreased compared to before. It is suggested that the hyperthyroid state caused by GD may promote the secretion of GH and IGF-1. And when the thyroid hormone levels decreased by using ATD, GH and IGF-1 levels also decreased.

Of the 11 patients included, six patients (cases 1, 5, 6, 7, 8, and 10) were not treated with ATD for GD during the disease and received only SSA and TSS treatment for GHPA. Among them, case 5 showed a significant decrease in GH/IGF-1 levels after SSA, as well as an improvement in thyroid function compared with that before SSA, as shown in Table 1. Later, the patient underwent TSS treatment at another hospital, but did not have thyroid function rechecked after surgery and was lost to follow-up. Cases 1, 6, 7, 8, and 10 were initially treated with SSA and had a mild decrease in their GH/IGF-1 levels compared to before, as well as thyroid function showing improvement in hyperthyroidism. As a result, the anesthesia risk was reduced, and they underwent TSS to successfully remove the tumor. Postoperative reexamination of pituitary target gland function indicated a significant decrease in GH/IGF-1, accompanied by a further decrease in thyroid function, as shown in Table 1. It shows that even if hyperthyroidism is not treated with ATD, the hyperthyroid state due to GD improved with a significant decrease in GH and IGF-1 levels after TSS or SSA for GHPA.

### Literature review

The PubMed/Medline electronic databases were searched for articles published before May 1, 2023, applying the following search terms: growth hormone-secreting pituitary adenoma, acromegaly, and Graves' disease. The criteria for papers to be included in this study were as follows: (1) confirmed diagnosis of GHPA and GD; (2) studies published in English; and (3) exclusion of reviews, expert opinions, or clinical guidelines. A total of seven original articles were included, which contained three males and four females, as shown in Table 2. All of the patients presented with acromegaly and thyrotoxicosis, and some of the patients were combined with proptosis. Treatments included TSS, bromocriptine, or cabergoline for GHPA, and ATD or 131I for GD.

**Table 2:** Cases of GHPA combined with GD in the published literature.

Author	Year	Country	Age (years)/ gender	Clinical manifestations	GH (ng/mL)	IGF-1 (ng/mL)	FT3 (pmol/L)	FT4 (pmol/L)	TSH (μIU/mL)	TRAb (IU/L)	Dmax of pituitary adenoma (mm)	Treatment
Hamilton (10)	1972	America	53/F	Signs of hyperthyroidism, proptosis, acromegaly	25	NA	NA	50.7	8.1	NA	NA	ATD, proton beam
Demura (11)	1984	Japan	44/M	Acral enlargement, perspiration	17.8	NA	NA	NA	<1	NA	NA	Hardy’s operation, ATD, bromocriptine
Shimatsu (8)	1990	Japan	30/M	Palpitations, acral enlargement	19.5	452	NA	135	<0.03	NA	NA	ATD, 131I, TSS
Hussein (12)	2005	Australia	32/M	Headache, weight loss, loss of libido	13.5	NA	NA	58.8	<0.04	19	15	Cabergoline, ATD
Tachibana (13)	2012	Japan	54/F	General fatigue, acromegalic features, mild proptosis	7.75	340	5.88	13	0.005	53.8%	NA	ATD, TSS
Di Cerbo (9)	2017	Italy	50/F	Signs of hyperthyroidism, acromegaly	26.0	615	44.5	77.6	undetectable	1120% of the basal	8	ATD, TSS
Halloul (14)	2021	Tunisia	64/F	Signs of hyperthyroidism, acromegaly	NA	800	NA	32.3	<0.05	NA	12	TSS, ATD

ATD, antithyroid drugs; Dmax, maximum diameter; F, female; FT3, free triiodothyronine; FT4, free thyroxine; GHPA**,**growth hormone-secreting pituitary adenoma; GD, Graves’ disease, GH, growth hormone; IGF-1, insulin-like growth factor 1; M, male; TSH, thyroid stimulating hormone; TRAb, thyroid stimulating hormone receptor antibodies; TSS, transsphenoidal surgery.

Shimatsu (8) studied the dynamics of GH secretion in a patient with acromegaly combined with GD during treatment with ATD and radioactive iodine, and the result showed that spontaneous secretion of tumor GH was stimulated in the hyperthyroid state. It suggests that the coexistence of GD exacerbates the severity of acromegaly. Di Cerbo (9) reported a case of acromegaly combined with GD who was initially treated with methimazole without significant improvement in hyperthyroidism. Owing to severe symptoms and signs of excessive secretion of GH/IGF-1, SSA treatment was initiated, and a few months later, the patient was treated with TSS. Postoperatively the patient also showed significant improvement in all indicators of GD activity. Hamilton (10) described a 53-year-old woman with combined toxic goiter and acromegaly who was cured of acromegaly by pituitary proton beam therapy and whose hyperthyroidism disappeared without other treatment. These two cases showed that the disease activity of acromegaly will also affect GD and that acromegaly in remission with treatment leads to improvement in the activity index, signs, and symptoms of GD. Combined with the cases we have reported above, it shows that the coexistence of GHPA and GD can complicate the diagnosis and treatment of the disease.

However, Demura (11) reported a 44-year-old man treated with Hardy's surgery for acromegaly who had normal thyroid function before surgery and developed hyperthyroidism and suppressed TSH immediately after surgery, consistent with GD. Propylthiouracil was effective in the treatment of hyperthyroidism. After he achieved normal thyroid function, plasma GH levels were also elevated. But the exact mechanism of the pathogenic effect of surgical stress on the hypothalamic-pituitary-thyroid axis is not known. Hussein (12) reported a case of a pituitary tumor secreting both GH and PRL in combination with GD, initially presenting with progressive headache, weight loss, and hypogonadism, in which thyroid hormone, GH, and PRL levels returned to normal, and the size of the pituitary mass was markedly reduced, after treatment with cabergoline and carbimazole.

In addition, the coexistence of other diseases in patients with GD combined with GHPA also complicates the treatment of the disease. Tachibana (13) reported a patient with GD and acromegaly who had primary hyperparathyroidism and underwent total parathyroidectomy and thyroidectomy, and who developed severe hypocalcemia induced by postsurgical hypoparathyroidism and hungry bone syndrome. Halloul (14) described a 64-year-old female patient presenting with signs of hyperthyroidism and an imbalance of her diabetes mellitus. On physical examination, she had facial features of acromegaly. Biochemical testing confirmed the suspicion of acromegaly and GD, and her MRI of the pituitary gland showed a large adenoma and an empty sella. The patient underwent successful TSS and her hyperthyroidism resolved with ATD.

## Discussion

### Effect of Graves’ hyperthyroidism on GH/IGF-1 secretion

The relationship between thyroid function and the GH–IGF-1 axis has been the subject of many reports, but previous studies have been unable to reach consistent conclusions regarding the relationship between serum FT3, FT4, or TSH and GH/IGF-1 levels in patients with GD. Some studies have shown a negative correlation between GH/IGF-1 levels and thyroid hormone levels in GD. In cats with hyperthyroidism, serum IGF-1 concentrations increased significantly after ATD treatment, and IGF-1 levels were negatively correlated with FT4 levels after diagnosis and treatment (15). Martin reported that nearly one-fifth of newly diagnosed GD patients had IGF-1 deficiency. Besides, IGF-1 deficiency is associated with more severe FT3 hyperthyroidism (16).

In contrast, other researchers have suggested that GH/IGF-1 levels are positively correlated with thyroid hormone levels in patients with GD. In the case reported by Shimatsu (8), in the hyperthyroid and normal thyroid states, mean basal plasma GH levels were 16.5 ± 0.9 μg/L and 7.8 ± 0.7 μg/L, respectively. Basal GH levels were positively correlated with FT4 levels (r = 0.902). In the thyrotoxic state, 24-hour plasma GH and urinary GH excretion were increased. Another study (17) evaluated serum IGF-1 levels in 30 patients with GD hyperthyroidism (HY group) and patients with normal thyroid function (EU group). HY group patients were treated with ATD as clinically indicated, while EU group patients were not treated with any drugs. The results showed that serum IGF-1 levels were significantly higher in HY group patients than in EU group patients at baseline and at 6 months of follow-up. At baseline, serum IGF-1 levels were positively correlated with FT4 (*β* = 29.02, *P* = 0.002) and negatively correlated with TSH (*β* = −31.46, *P* = 0.042) and logTSH (*β* = −29.04, *P* = 0.007) in both HY group and EU group patients. Hussein's (12) study showed a positive correlation between IGF-1 levels and TRAb at baseline, but we did not find a correlation between GH or IGF-1 levels and TRAb at baseline.

Our findings suggest that in patients with GHPA combined with GD, GD causes hyperthyroidism and after ATD was applied, GH/IGF-1 levels were significantly lower than before. It indicates that hyperthyroidism promotes the secretion of GH/IGF-1, which is detrimental to the biochemical control of GHPA. Therefore, patients with GHPA combined with GD should be treated with ATD preoperatively for thyrotoxicosis to restore thyroid function to normal levels, which will not only help reduce the risk of anesthesia but also facilitate the biochemical control of GHPA postoperatively.

The mechanism of altered GH secretion in patients with hyperthyroidism is not fully illuminated. Iranmanesh reported an almost four-fold increase in 24-h GH secretion rate in hyperthyroid patients, which may be related to increased endogenous growth hormone-releasing hormone (GHRH) secretion (18). It has also been suggested that thyroid hormones may regulate GH secretion by acting directly on anterior pituitary cells or by regulating the release of hypothalamic GHRH and somatostatin (19, 20).

### The role of GH/IGF-1 in the pathogenesis of GD

Recent studies have shown that the coexistence of GHPA exacerbates GD, while GD improves after treatment of GHPA with TSS or SSA. In the case of acromegaly combined with GD reported by Di Cerbo (9), GD activity paralleled acromegaly activity as assessed by the ability of the patient's IgG to block TSH binding and induce the production of cyclic adenosine monophosphate (cAMP), and the activity index and symptom of GD improved after surgical remission of acromegaly, confirming the hypothesis that the GH−IGF-1 axis may play an important role in maintaining GD activity and exacerbating the clinical course of GD.

The action of somatostatin is mediated by five somatostatin receptor (SSTR) subtypes, including SSTR1-5 (21). Studies have shown that SSTR2 is overexpressed in diseased thyroid tissues, while SSTR1 and 5 are present in all normal thyroid tissues and are the main receptors in malignant tissues, whereas in benign tissues, especially in GD tissues, the expression of SSTR1 and 5 is limited (22). SSA is commonly used in the treatment of patients with acromegaly and exerts central and direct effects on thyroid function. At the pituitary level, SSA inhibits thyrotropin cell proliferation and TSH secretion and can be used to treat TSH-secreting pituitary adenomas (23). At the thyroid level, SSA affects thyroid function through SSTRs expressed on thyroid cells (22). Nomoto (24) reported a woman with acromegaly combined with autonomously functioning thyroid nodules with typical physical features of acromegaly and hyperthyroidism with TSH suppression, who returned to normal IGF-1 levels and thyroid function after treatment with octreotide. In our study, case 5 showed a significant decrease in GH/IGF-1 and an improvement in hyperthyroidism after receiving SSA treatment alone. Similarly, after the application of SSA, cases 1, 6, 7, 8, and 10 also showed a mild decrease in thyroid hormone levels, and after undergoing TSS to remove the tumor, there was a significant decrease in GH/IGF-1 levels, accompanied by a further significant decrease in thyroid hormone levels. Therefore, in the treatment of GHPA combined with GD, SSA may affect thyroid function through direct action on the thyroid gland, in addition to its role in lowering GH/IGF-1 levels.

These results show that in the absence of ATD treatment for GD hyperthyroidism, patients with GHPA combined with GD treated with TSS or SSA experienced a significant decrease in thyroid hormones along with a decrease in GH/IGF-1 levels. It is suggested that the coexistence of GHPA aggravates the severity of GD, and the activity index, signs, and symptoms of GD improve after a decrease in GH/IGF-1 levels by surgery or pharmacological treatment. Therefore, when GHPA is combined with GD, aggressive biochemical control in patients with GHPA can contribute to the improvement of hyperthyroidism in GD.

Several studies have confirmed the important role of GH in the pathogenesis of GD, which is involved in the immune regulation of cells by binding to receptors on the cell membrane. The growth hormone receptor belongs to the cytokine receptor superfamily and is abundantly expressed in B lymphocytes and less frequently in T lymphocytes (25, 26). Evidence from previous studies suggests that GH enhances T cell proliferation and *in vitro* human B cell immunoglobulin production and proliferation, and this effect is not mediated by IGF-1 (27, 28), suggesting that GH itself may stimulate TRAb production by B lymphocytes.

Besides, many studies have also elucidated the effects of IGF-1/insulin like growth factor 1 receptor (IGF-1R) signaling pathway activation on immune function. IGF-1 affects the development and function of T and B lymphocytes and thyroid cells by binding the tyrosine kinase IGF-1R (29). *In vitro* studies have shown that IGF-1 synergizes the TSH-induced thyroid cell growth activation pathway independent of the TSH/cAMP/protein kinase cascade reaction. Tramontano's study demonstrated the relationship between IGF-1R and thyroid stimulating hormone receptor (TSHR), showing that IGF-1, TSH, and Graves' immunoglobulin have synergistic and dose-dependent effects on DNA synthesis and thyroid cell proliferation (30, 31). In conclusion, GH/IGF-1 may act through the following mechanisms: (1) by stimulating T-cell proliferation and T-cell infiltration of the thyroid to produce inflammatory cytokines; (2) by stimulating B-cell immunoglobulin production and proliferation, and (3) by promoting the post-receptor pathway directly in thyroid cells. Therefore, the coexistence of both GHPA and GD is considered a possible *in vivo* model to study the role of the GH/IGF-1 axis on the immune system in patients with GD.

Of the 11 patients included in this study, case 8 had mild proptosis, which was not treated with ATD, after GH/IGF-1 levels decreased after TSS and SSA treatment, the symptoms of proptosis improved. In previous reports in the literature, patients with GHPA combined with GD have also been described as having symptoms of proptosis. In the case reported by Tachibana (13), the patient's proptosis improved after combined ATD and TSS treatment. In the case reported by Hamilton (10), the patient was treated with pituitary proton beam therapy only, and with the decrease of GH/IGF-1, the patient's symptoms of proptosis were reduced. Another case report describes a patient with adult growth hormone deficiency who presented with bilateral lacrimal gland and eyelid swelling 1 month after initiation of GH therapy and was diagnosed with GO, presenting with mild thyrotoxicosis and elevated TRAb levels. Clinical activity scores for GO decreased after discontinuation of GH injections. The combination of glucocorticoids and radiation therapy further improved GO, suggesting that GH/IGF-1 signaling is a risk factor for the development of GO (32). IGF-1R is expressed at higher levels in the orbital connective tissue of GO patients than in normal tissue (33). Teprotomumab is a fully human monoclonal IGF-1R antagonist, and by inhibiting the IGF-1R/TSHR signaling pathway, teprotomumab reduces pro-inflammatory cytokine production, hyaluronan secretion, and orbital fibroblast activation in GO patients, and is currently approved for the treatment of GO (34). Therefore, in patients with GHPA combined with GD, controlling GH/IGF-1 levels not only helps to avoid further worsening of hyperthyroidism due to GD but also helps to reduce the clinical activity of GO.

## Conclusion

The combination of GHPA with autoimmune thyroid disease, especially GD, although rare, should not be ignored. GD hyperthyroidism affects the synthesis and secretion of GH/IGF-1. Preoperative treatment of GD hyperthyroidism with ATD not only helps to reduce the risk of anesthesia but also helps to achieve better biochemical control of GHPA after surgery. On the other hand, the coexistence of GHPA can also aggravate the hyperthyroidism caused by GD and may even induce or aggravate GO, while the hyperthyroidism of GD improves with the decrease of GH/IGF-1 after TSS or SSA treatment, suggesting that the GH–IGF-1 axis promotes growth, thyroid function, and thyroid hormone metabolism. Therefore, clinicians should pay attention to identifying the combination of GHPA with GD, which is important for the disease treatment and prognosis of patients.

## Declaration of interest

The authors have no conflicts of interest to declare.

## Funding

This research did not receive any specific grant from any funding agency in the public, commercial, or not-for-profit sector.

## Statement of ethics

The work described has been carried out following the principles of the Declaration of Helsinki and the study was approved by the Human Research Ethics Committee of Beijing Tiantan Hospital, Capital Medical University (approval number: KY2022-024-01). Written informed consent was obtained from all participants in the study.

## Data availability statement

All data generated or analyzed during this study are included in this article. Further inquiries can be directed to the corresponding author.

## Author contribution statement

Caiyan Mo collected the data and wrote the first draft, Tao Tong assisted in collecting the data, Ying Guo and Zheng Li provided writing guidance, and Liyong Zhong developed the research proposal and guided the writing. All the authors read and approved the final version of the manuscript.
